# Crystal structure of a three-dimensional neodymium(III) coordination polymer, [Nd_2_(H_2_O)_6_(glutarato)(SO_4_)_2_]_*n*_

**DOI:** 10.1107/S2056989022000159

**Published:** 2022-01-11

**Authors:** Saranphong Yimklan, Yothin Chimupala, Sutsiri Wongngam, Nippich Kaeosamut

**Affiliations:** a Department of Chemistry, Faculty of Science, Chiang Mai University, Chiang Mai, 50200, Thailand; b Department of Industrial Chemistry, Faculty of Science, Chiang Mai University, Chiang Mai, 50200, Thailand

**Keywords:** crystal structure, coordination polymer, lanthanide, glutarate

## Abstract

A three-dimensional coordination polymer, poly[hexa­aqua­(μ_4_-glutarato)bis(μ_3_-sulfato)­dineodymium(III)], [Nd_2_(glutarato)(SO_4_)_2_(H_2_O)_6_]_*n*_ (glutarato^2–^ = C_5_H_6_O_4_^2–^), **1**, consisting of cationic {Nd_2_(H_2_O)_6_(SO_4_)_2_}_*n*_^2*n*+^ layers linked by bridging glutarate ligands, was synthesized by the microwave-heating technique within few minutes. The crystal structure of **1** consists of two crystallographically independent *TPRS*-{Nd^III^O_9_} (*TPRS* is tricapped trigonal–prismatic geometry) units that form an edge-sharing dinuclear cluster inter­connected to neighbouring dimers by the μ_3_-SO_4_^2–^ anions, yielding a cationic two-dimensional {Nd_2_(H_2_O)_6_(SO_4_)_2_}_*n*_^2*n*+^ sheet. Adjacent cationic layers are then linked *via* the μ_4_-glutarato^2–^ ligands into a three-dimensional coordination network. Strong O—H⋯O hydrogen bonds are the predominant inter­action in the crystal structure.

## Abstract 2

A three-dimensional coordination polymer, poly[hexa­aqua­(μ_4_-glutarato)bis­(μ_3_-sulfato)­dineodymium(III))], [Nd_2_(H_2_O)_6_(glutarato)(SO_4_)_2_]_*n*_ (glutarato^2–^ = C_5_H_6_O_2_^2–^), consisting of cationic {Nd_2_(H_2_O)_6_(SO_4_)_2_}_*n*_^2*n*+^ layers linked by glutarate ligands, was synthesized by the microwave-heating technique.

## Chemical context

Coordination polymers (CPs) and metal–organic frameworks (MOFs) have attracted much attention because of the fascin­ating tuneability of their mol­ecular architectures and functionalities that helps to adjust their properties for applications in different areas such as in sensing and magnetism, as well as catalysis. These properties are cooperatively provided by both the inorganic building units and the organic counterparts (Furukawa *et al.*, 2013 ▸). Across the periodic table, the *not-so-rare* earth lanthanides (*Ln*) have become one of the promising choices for such materials because of their robust *Ln*—O bonds, versatile coordination geometries and high thermal stability with exotic properties, including photoluminescence and adaptive active sites for catalysis (Pagis *et al.*, 2016 ▸). On the other hand, the flexibility of the organic linkers, such as aliphatic polycarboxyl­ates, can also diversify the structural architecture that sometimes defines the macroscopic properties of the materials (Kim *et al.*, 2017 ▸).

Herein, we report a microwave synthesis of a new three-dimensional coordination polymer, [Nd_2_(H_2_O)_6_(glutarato)(SO_4_)_2_]_*n*_ (**1**). The crystal structure reveals that the glutarates act as bridging ligands binding the cationic {Nd_2_(H_2_O)_6_(SO_4_)_2_}_*n*_^2*n*+^ sheets into a three-dimensional network.

## Structural commentary

**Figure 1:**
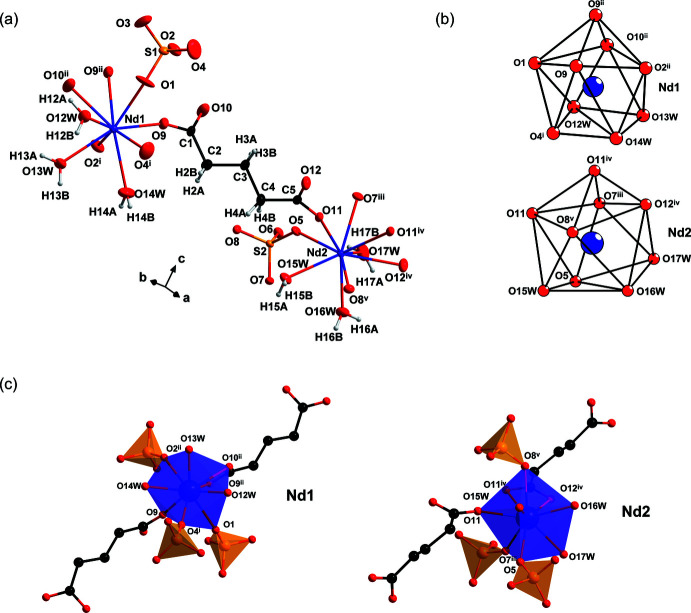
Graphical representations of (*a*) an extended asymmetric unit of **1** drawn with 50% probability ellipsoids, (*b*) coordination geometries of Nd1 (top) and Nd2 (bottom) and (*c*) coordination environment of Nd1 (left) and Nd2 (right). [Symmetry codes: (i) −*x* + 2, −*y*, −*z*; (ii) −*x* + 2, *y -* 1/2, −*z* − 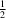 ; (iii) *x*, −*y* + 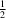 , *z* + 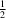 ; (iv) −*x* + 1, −*y* + 1, −*z*; (v) *x*, −*y* + 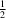 , *z* − 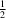 ; (vi) −*x* + 2, *y* + 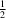 , −*z* − 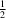 .]

The coordination network **1**, [Nd_2_(H_2_O)_6_(glutarato)(SO_4_)_2_]_*n*_ crystallizes in the monoclinic *P*2_1_/*c* space group. There are two crystallographically independent Nd^III^ cations (Nd1 and Nd2), two sulfate anions, and six coordinated water mol­ecules in the asymmetric unit, as illustrated in Fig. 1 ▸. 
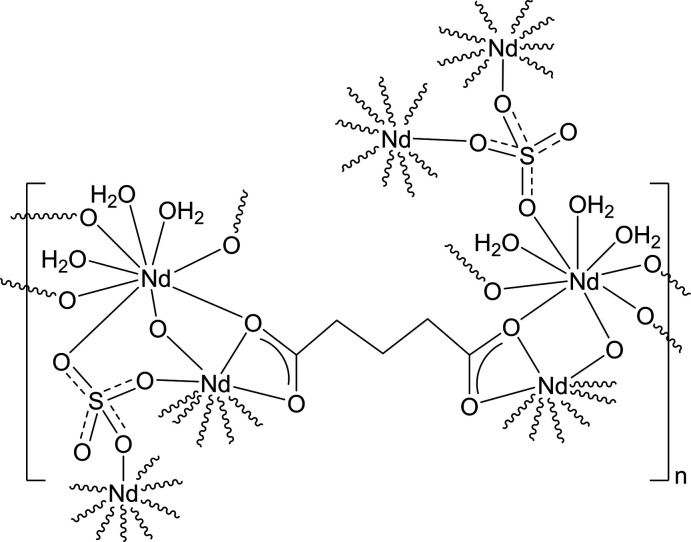


**Figure 2:**
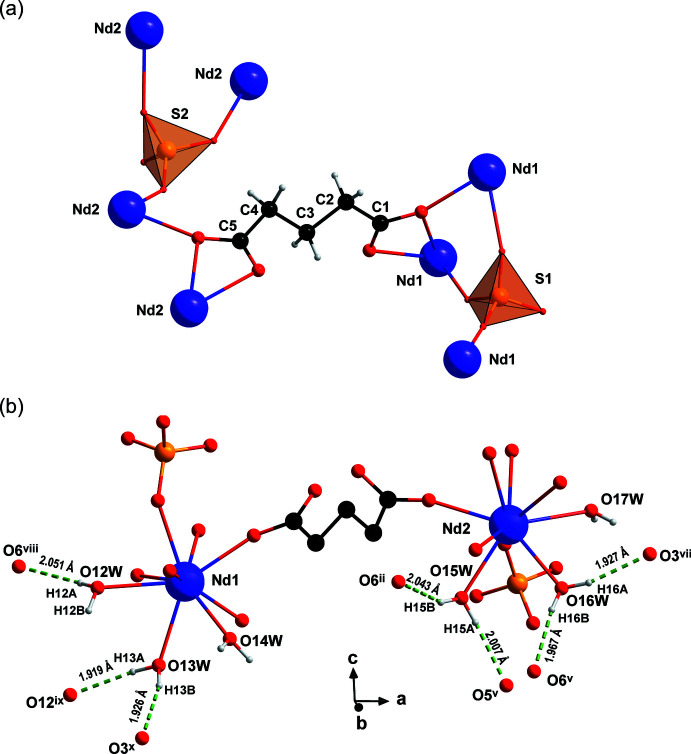
Depictions of (*a*) the coordination modes of the glutarate and the sulfate ligands in **1** and (*b*) seven of the eleven crystallographically independent hydrogen bonds (dashed green lines) with bond distances. [Symmetry codes: (i) −*x* + 2, −*y*, −*z*; (ii) −*x* + 2, *y* − 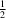 , −*z* − 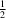 ; (iii) *x*, −*y* + 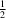 , *z* + 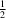 ; (iv) −*x* + 1, −*y* + 1, −*z*; (v) *x*, −*y* + 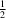 , *z* − 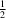 ; (vi) −*x* + 2, *y* + 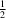 , −*z* − 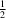 ; (vii) *x* + 1, −*y* + 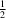 , *z* − 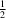 ; (viii) *x* − 1, *y*, *z*; (ix) −*x* + 1, *y* + 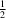 , −*z* − 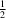 ; (x) *x*, *y*, *z* − 1.]

Both Nd^III^ cations are nine-coordinated to O atoms from one bridging glutarate^2−^, two chelating glutarate^2−^, two chelating sulfate anions and three coordinated H_2_O, adopting a distorted tricapped trigonal–prismatic geometry, *TPRS*-{Nd^III^O_9_} (*see* Fig. 1 ▸ *b*), forming an edge-sharing dinuclear unit with its symmetry-related Nd^III^O_9_ polyhedron. The Nd^III^—O bond distances are in the range of 2.383 (2)–2.785 (2) Å, which are reasonable and comparable to those reported for other Nd^III^ coordination polymers such as [Nd(H_2_O)_4_(glutarato)]Cl (Hussain *et al.*, 2015 ▸), [Nd(H_2_O)_4_(glutarato)]Cl·2H_2_O (Leg­end­ziewicz *et al.*, 1999 ▸) and [Nd_2_(H_2_O)_2_(glutarato)]·2H_2_O (Głowiak *et al.*, 1986 ▸). In contrast to the above-mentioned coordination polymers, [Nd(glutarato)(H_2_O)_4_]Cl (Hussain *et al.*, 2015 ▸) and [Nd(glutarato)(H_2_O)_4_]Cl·2H_2_O (Legendziewicz *et al.*, 1999 ▸) consisting of cationic {Nd(H_2_O)_x_(glutarato)}*_n_^n^* ^+^ (*x* = 2, 4) subunits compensated by uncoordinated chloride anions, each of the tetra­hedral SO_4_^2–^ ligands in **1** links three adjacent Nd^III^ atoms, forming a neutral two-dimensional network of [Nd_2_(H_2_O)_6_(glutarato)(SO_4_)_2_]_*n*_. The S—O bond distances are in the range 1.449 (3)–1.485 (2) Å, with O—S—O angles ranging from 107.78 (16) to 111.67 (15)°. The flexible glutarate linker exhibits a (μ_4_-κ^2^ *O*:κO′:κ^2^O′′:κ*O*′′′ coordination mode with an *anti*–*anti* conformation as depicted in Fig. 2 ▸ *a*. There are six crystallographically independent water mol­ecules completing the coordination sites of the two Nd^III^ atoms (three H_2_O mol­ecules for each Nd^III^ atom, Fig. 2 ▸ *b*).

## Supra­molecular features

**Figure 3:**
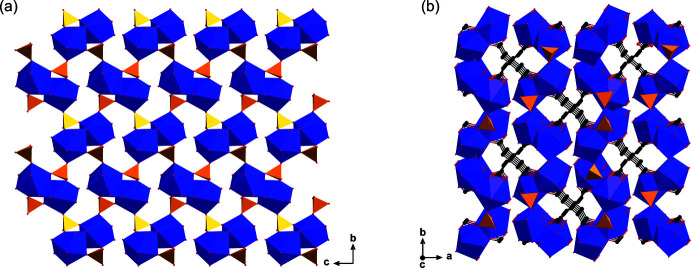
Views of (*a*) the [Nd_2_(H_2_O)_6_(SO_4_)_2_]_*n*_^2*n*+^ sheet and (*b*) the three-dimensional framework of **1**.

The polymeric structure of **1** can be described as a three-dimensional non-porous framework, which is constructed from edge-sharing *TPRS*-{Nd^III^O_9_} polyhedra linked through sulfate anions, acting as tritopic inorganic linkers, into a cationic [Nd_2_(H_2_O)_6_(SO_4_)_2_]_*n*_^2*n*+^ sheets parallel to the (011) layers, as illustrated in Fig. 3 ▸ *a*. It is noteworthy that these sheets also contain large inorganic [Nd(SO_4_)]_4_ rings further stabilized by O—H⋯O hydrogen bonds between the water mol­ecules and sulfate anions (Table 1 ▸). Eventually, the final three-dimensional network is formed by connecting these adjacent cationic sheets by the glutarate ligands (Fig. 3 ▸ *b*). This three-dimensional arrangement also features O—H⋯O hydrogen bonds between two water mol­ecules or between a water mol­ecule and oxygen atoms of the glutarate ligands (Fig. 2 ▸ *b*). In total, all but one hydrogen atom from the six crystallographically independent water mol­ecules are involved in hydrogen bonding (Table 1 ▸). Analysis of these hydrogen bonds revealed thirteen different first-order graph sets (Bernstein *et al.*, 1995 ▸) consisting of five rings and eight different chains.

## Database survey

A search of the Cambridge Structural Database (CSD, Version 5.42, update of September 2021; Groom *et al.*, 2016 ▸) confirms that no Nd^III^ coordination polymer containing both glutarate^2–^ and SO_4_^2–^ has been reported. However, several related polymeric structures, *viz. catena*-[(μ-penta­nedio­ato)tetra­aqua­neo­dym­ium chloride] (NEMXIP; Hussain *et al.*, 2015 ▸), *catena*-[(μ_4_-glutarato)tetra­aqua­dineodymium chloride dihydrate] (DIQZAE01; Marsh, 2005 ▸), *catena*-[bis­(μ_4_-pen­tane-1,5-dionato)(μ_2_-pentane-1,5-dionato)di­aqua­di­neo­dym­ium(III) tetra­hydrate] (FAQYUR; Legendziewicz *et al.*, 1999 ▸) and *catena*-[tris­(μ_3_-glutarato-*O*,*O*,*O′*,*O′′*,*O′′′*)diaqua­di­neodymium(III) dihydrate] (FAFGAU; Głowiak *et al.*, 1986 ▸), have been reported.

## Synthesis and crystallization

Complex **1** was synthesized by dissolving Nd_2_(SO_4_)_3_·8H_2_O (1 mmol, 0.721 g), glutaric acid (1 mmol, 0.132 g), and 4,4′-bi­pyridine (1 mmol, 0.156 g) in 40.0 mL of deionized water under ambient conditions. The solution was transferred into an open glass reactor and then irradiated by microwaves (800 W) for 10 minutes. The solution was let to cool to ambient temperature. Pale-purple block-shaped crystals crystallized from the solution within a few minutes. FT–IR (ATR Mode, cm^−1^) of **1**: *ν_stretch_* (O—H) 3364, *ν_stretch_* (C—H) 2990, *ν_as_* (COO^−^) 1531, *ν_s_* (COO^−^) 1430, *δ*(O—H) 1355, *ν_s_* (S—O) 1101, *ν_s_* (S—O) 1077, *ν_s_* (SO_4_^2–^) 596.

## Refinement

Crystal data, data collection and structure refinement details are summarized in Table 2 ▸. Carbon-bound H atoms were positioned geometrically (C—H = 0.97 Å) and constrained using the riding-model approximation with *U*_iso_(H) = 1.2*U*_eq_(C). The H atoms from the water mol­ecules were located in the residual electron-density map, and where necessary, refined with distance and angle restraints or riding on the parent oxygen atom.

## Supplementary Material

Crystal structure: contains datablock(s) I. DOI: 10.1107/S2056989022000159/jq2009sup1.cif

Structure factors: contains datablock(s) I. DOI: 10.1107/S2056989022000159/jq2009Isup2.hkl

Supporting information file. DOI: 10.1107/S2056989022000159/jq2009Isup3.mol

CCDC reference: 2107848

Additional supporting information:  crystallographic information; 3D view; checkCIF report

## Figures and Tables

**Table 1:** Hydrogen-bond geometry (Å, °)

*D*—H⋯*A*	*D*—H	H⋯*A*	*D*⋯*A*	*D*—H⋯*A*
O16*W*—H16*A*⋯O3^i^	0.85	1.91	2.709 (4)	155
O16*W*—H16*B*⋯O6^ii^	0.85	1.94	2.774 (3)	165
O17*W*—H17*A*⋯O12*W* ^iii^	0.81 (2)	2.38 (4)	2.966 (4)	130 (4)
O17*W*—H17*B*⋯O3^i^	0.84 (2)	2.09 (2)	2.903 (4)	165 (4)
O12*W*—H12*A*⋯O6^iv^	0.85	2.10	2.825 (4)	143
O12*W*—H12*B*⋯O17*W* ^v^	0.85	2.07	2.904 (4)	166
O13*W*—H13*A*⋯O12^vi^	0.85	1.95	2.733 (3)	153
O13*W*—H13*B*⋯O3^vii^	0.85	1.99	2.745 (4)	148
O14*W*—H14*A*⋯O13*W* ^viii^	0.85 (2)	2.13 (2)	2.966 (4)	165 (4)
O18—H18*A*⋯O6^ix^	0.83 (2)	2.03 (2)	2.826 (3)	160 (3)
O18—H18*B*⋯O5^ii^	0.81 (2)	2.00 (2)	2.805 (3)	170 (4)

Symmetry codes: (i) x+1, -y+{\script{1\over 2}}, z-{\script{1\over 2}}; (ii) x, -y+{\script{1\over 2}}, z-{\script{1\over 2}}; (iii) x+1, y, z; (iv) x-1, y, z; (v) x-1, -y+{\script{1\over 2}}, z-{\script{1\over 2}}; (vi) -x+1, y+{\script{1\over 2}}, -z-{\script{1\over 2}}; (vii) x, y, z-1; (viii) -x+1, -y+1, -z-1; (ix) -x+2, y-{\script{1\over 2}}, -z-{\script{1\over 2}}.

**Table 2:** Experimental details

Crystal data	
Chemical formula	[Nd_2_(C_5_H_6_O_4_)(SO_4_)_2_(H_2_O)_6_]
*M*_r_	718.79
Crystal system, space group	Monoclinic, *P*2_1_/*c*
Temperature (K)	293
*a*, *b*, *c* (Å)	15.5461 (1), 12.6621 (1), 8.8883 (1)
β (°)	95.287 (1)
*V* (Å^3^)	1742.19 (3)
*Z*	4
Radiation type	Mo *K*α
μ (mm^−1^)	6.23
Crystal size (mm)	0.2 × 0.2 × 0.2

Data collection	
Diffractometer	SuperNova, Single source at offset/far, HyPix3000
Absorption correction	Multi-scan (*CrysAlis PRO*; Agilent, 2014 ▸)
*T*_min_, *T*_max_	0.448, 1.000
No. of measured, independent and observed [*I* > 2σ(*I*)] reflections	38711, 3830, 3482
*R*_int_	0.067
(sin θ/λ)_max_ (Å^−1^)	0.648

Refinement	
*R*[*F* ^2^ > 2σ(*F* ^2^)], *wR*(*F* ^2^), *S*	0.022, 0.045, 1.08
No. of reflections	3830
No. of parameters	268
No. of restraints	9
H-atom treatment	H atoms treated by a mixture of independent and constrained refinement
Δρ_max_, Δρ_min_ (e Å^−3^)	0.62, −0.88

Computer programs: *CrysAlis PRO* (Agilent, 2014 ▸), *SHELXT2018/2* (Sheldrick, 2015*a* ▸), *SHELXL2018/3* (Sheldrick, 2015*b* ▸) and *OLEX2* (Dolomanov *et al.*, 2009 ▸).

## supplementary crystallographic information

### Crystal data

**Table:**

[Nd_2_(C_5_H_6_O_4_)(SO_4_)_2_(H_2_O)_6_]	*F*(000) = 1376
*M**_r_* = 718.79	*D*_x_ = 2.740 Mg m^−^^3^
Monoclinic, *P*2_1_/*c*	Mo *K*α radiation, λ = 0.71073 Å
*a* = 15.5461 (1) Å	Cell parameters from 25026 reflections
*b* = 12.6621 (1) Å	θ = 2.1–27.3°
*c* = 8.8883 (1) Å	µ = 6.23 mm^−^^1^
β = 95.287 (1)°	*T* = 293 K
*V* = 1742.19 (3) Å^3^	Block, clear light violet
*Z* = 4	0.2 × 0.2 × 0.2 mm

### Data collection

**Table:**

SuperNova, Single source at offset/far, HyPix3000 diffractometer	3830 independent reflections
Radiation source: micro-focus sealed X-ray tube, SuperNova (Mo) X-ray Source	3482 reflections with *I* > 2σ(*I*)
Mirror monochromator	*R*_int_ = 0.067
ω scans	θ_max_ = 27.4°, θ_min_ = 2.1°
Absorption correction: multi-scan (CrysAlisPro; Agilent, 2014)	*h* = −20→19
*T*_min_ = 0.448, *T*_max_ = 1.000	*k* = −16→16
38711 measured reflections	*l* = −11→11

### Refinement

**Table:**

Refinement on *F*^2^	Secondary atom site location: difference Fourier map
Least-squares matrix: full	Hydrogen site location: mixed
*R*[*F*^2^ > 2σ(*F*^2^)] = 0.022	H atoms treated by a mixture of independent and constrained refinement
*wR*(*F*^2^) = 0.045	*w* = 1/[σ^2^(*F*_o_^2^) + (0.0106*P*)^2^ + 1.2155*P*] where *P* = (*F*_o_^2^ + 2*F*_c_^2^)/3
*S* = 1.08	(Δ/σ)_max_ = 0.003
3830 reflections	Δρ_max_ = 0.62 e Å^−^^3^
268 parameters	Δρ_min_ = −0.88 e Å^−^^3^
9 restraints	Extinction correction: SHELXL2018/3 (Sheldrick 2015b), Fc^*^=kFc[1+0.001xFc^2^λ^3^/sin(2θ)]^-1/4^
Primary atom site location: structure-invariant direct methods	Extinction coefficient: 0.00033 (5)

### Special details

**Table:**

Geometry. All esds (except the esd in the dihedral angle between two l.s. planes) are estimated using the full covariance matrix. The cell esds are taken into account individually in the estimation of esds in distances, angles and torsion angles; correlations between esds in cell parameters are only used when they are defined by crystal symmetry. An approximate (isotropic) treatment of cell esds is used for estimating esds involving l.s. planes.
Refinement. The structure of 1 was solved in the space group P21/c (No. 14) using direct methods in the SHELXT (Sheldrick, 2015a) structure-solution program and refined by full-matrix least-squares minimization on F2 using SHELXL 2018/3 (Sheldrick, 2015b).

### Fractional atomic coordinates and isotropic or equivalent isotropic displacement parameters (Å^2^)

**Table:**

	*x*	*y*	*z*	*U*_iso_*/*U*_eq_	
Nd2	1.07588 (2)	0.09859 (2)	−0.12226 (2)	0.01075 (6)	
Nd1	0.42661 (2)	0.43378 (2)	−0.18797 (2)	0.01254 (6)	
S2	1.05205 (6)	0.38265 (6)	−0.21038 (9)	0.01194 (18)	
S1	0.41004 (6)	0.35878 (6)	0.21539 (9)	0.01539 (19)	
O11	0.93262 (15)	0.07269 (16)	−0.0510 (2)	0.0157 (5)	
O6	1.11665 (15)	0.45391 (16)	−0.1313 (2)	0.0180 (5)	
O5	1.04359 (16)	0.28751 (16)	−0.1153 (2)	0.0177 (5)	
O8	0.96805 (15)	0.43635 (16)	−0.2326 (2)	0.0155 (5)	
O16W	1.16897 (17)	0.0982 (2)	−0.3338 (3)	0.0265 (6)	
H16A	1.222796	0.084873	−0.321649	0.040*	
H16B	1.152057	0.070890	−0.418758	0.040*	
O17W	1.21843 (18)	0.1988 (2)	−0.0366 (3)	0.0321 (7)	
H17A	1.218 (3)	0.2626 (15)	−0.044 (5)	0.048*	
H17B	1.259 (2)	0.177 (3)	−0.083 (4)	0.048*	
O12	0.80870 (15)	0.03120 (18)	0.0300 (3)	0.0220 (6)	
O12W	0.27085 (16)	0.37303 (19)	−0.2316 (3)	0.0258 (6)	
H12A	0.237228	0.422979	−0.210018	0.039*	
H12B	0.258297	0.362463	−0.325853	0.039*	
O9	0.56170 (17)	0.41489 (18)	−0.0404 (3)	0.0236 (6)	
O2	0.48805 (16)	0.42067 (18)	0.2617 (3)	0.0247 (6)	
O13W	0.36280 (16)	0.5016 (2)	−0.4426 (3)	0.0283 (6)	
H13A	0.309153	0.513771	−0.439262	0.042*	
H13B	0.364813	0.453202	−0.508542	0.042*	
O10	0.67742 (18)	0.4309 (2)	0.1157 (3)	0.0335 (7)	
O14W	0.5138 (2)	0.3668 (2)	−0.4039 (3)	0.0425 (8)	
H14A	0.545 (3)	0.400 (3)	−0.463 (4)	0.064*	
H14B	0.498 (3)	0.313 (2)	−0.451 (5)	0.064*	
O1	0.39367 (18)	0.3531 (2)	0.0516 (3)	0.0348 (7)	
C4	0.8121 (2)	0.1900 (3)	−0.1158 (4)	0.0213 (8)	
H4A	0.776496	0.167837	−0.205583	0.026*	
H4B	0.856900	0.236004	−0.147741	0.026*	
C1	0.6402 (2)	0.3905 (3)	−0.0018 (4)	0.0178 (8)	
C5	0.8539 (2)	0.0942 (2)	−0.0408 (4)	0.0148 (8)	
C2	0.6882 (3)	0.3205 (3)	−0.1001 (4)	0.0297 (10)	
H2A	0.715651	0.364043	−0.171662	0.036*	
H2B	0.647082	0.274906	−0.157345	0.036*	
C3	0.7565 (2)	0.2524 (3)	−0.0148 (4)	0.0249 (9)	
H3A	0.728270	0.203229	0.048365	0.030*	
H3B	0.793796	0.297267	0.051188	0.030*	
O18	0.97596 (18)	0.14621 (19)	−0.3490 (3)	0.0206 (6)	
H18A	0.945 (2)	0.095 (2)	−0.376 (4)	0.031*	
H18B	0.992 (2)	0.172 (3)	−0.425 (3)	0.031*	
O7	1.08197 (15)	0.34921 (17)	−0.3553 (2)	0.0160 (5)	
O4	0.4228 (2)	0.2533 (2)	0.2767 (3)	0.0447 (8)	
O3	0.33686 (18)	0.4071 (2)	0.2790 (3)	0.0431 (8)	

### Atomic displacement parameters (Å^2^)

**Table:**

	*U*^11^	*U*^22^	*U*^33^	*U*^12^	*U*^13^	*U*^23^
Nd2	0.01192 (11)	0.01076 (10)	0.00957 (10)	0.00054 (7)	0.00104 (8)	0.00014 (7)
Nd1	0.01289 (12)	0.01314 (11)	0.01148 (10)	0.00009 (7)	0.00057 (8)	0.00022 (7)
S2	0.0158 (5)	0.0112 (4)	0.0089 (4)	0.0013 (3)	0.0013 (3)	0.0007 (3)
S1	0.0187 (5)	0.0147 (4)	0.0133 (4)	−0.0029 (4)	0.0047 (4)	0.0003 (3)
O11	0.0119 (13)	0.0187 (12)	0.0161 (13)	0.0037 (10)	0.0000 (11)	−0.0004 (10)
O6	0.0206 (14)	0.0169 (12)	0.0154 (12)	−0.0016 (11)	−0.0037 (11)	−0.0019 (10)
O5	0.0272 (15)	0.0134 (12)	0.0128 (12)	0.0019 (11)	0.0028 (11)	0.0042 (9)
O8	0.0177 (14)	0.0140 (12)	0.0152 (12)	0.0039 (10)	0.0036 (11)	0.0022 (9)
O16W	0.0175 (15)	0.0443 (17)	0.0177 (14)	−0.0005 (13)	0.0022 (12)	−0.0048 (12)
O17W	0.0298 (18)	0.0261 (15)	0.0389 (18)	−0.0074 (14)	−0.0057 (14)	0.0082 (14)
O12	0.0154 (14)	0.0191 (13)	0.0313 (14)	0.0002 (11)	0.0010 (12)	0.0079 (11)
O12W	0.0194 (15)	0.0312 (15)	0.0261 (14)	−0.0007 (12)	−0.0024 (12)	−0.0030 (12)
O9	0.0178 (15)	0.0285 (14)	0.0241 (14)	0.0071 (11)	0.0006 (12)	0.0010 (11)
O2	0.0240 (16)	0.0300 (14)	0.0193 (14)	−0.0128 (12)	−0.0021 (12)	0.0040 (11)
O13W	0.0205 (15)	0.0439 (17)	0.0201 (13)	0.0049 (13)	0.0003 (12)	−0.0037 (12)
O10	0.0281 (17)	0.0360 (16)	0.0342 (16)	0.0110 (13)	−0.0090 (14)	−0.0181 (13)
O14W	0.061 (2)	0.0426 (19)	0.0262 (17)	0.0041 (17)	0.0190 (16)	−0.0004 (14)
O1	0.0471 (19)	0.0486 (17)	0.0084 (12)	−0.0274 (15)	0.0015 (12)	−0.0004 (12)
C4	0.020 (2)	0.0195 (19)	0.024 (2)	0.0058 (16)	0.0006 (16)	0.0038 (15)
C1	0.015 (2)	0.0190 (18)	0.0188 (19)	0.0056 (15)	−0.0017 (16)	−0.0020 (15)
C5	0.015 (2)	0.0144 (17)	0.0149 (18)	0.0013 (15)	−0.0009 (15)	−0.0031 (14)
C2	0.029 (2)	0.038 (2)	0.022 (2)	0.0139 (19)	−0.0004 (18)	−0.0049 (17)
C3	0.030 (2)	0.023 (2)	0.0209 (19)	0.0139 (17)	0.0010 (17)	−0.0023 (16)
O18	0.0267 (16)	0.0193 (14)	0.0155 (13)	−0.0062 (11)	−0.0003 (12)	0.0043 (11)
O7	0.0188 (14)	0.0185 (12)	0.0111 (11)	0.0050 (10)	0.0030 (10)	−0.0002 (9)
O4	0.057 (2)	0.0187 (15)	0.0566 (19)	−0.0073 (14)	−0.0032 (17)	0.0138 (14)
O3	0.0171 (16)	0.069 (2)	0.0430 (18)	0.0099 (14)	0.0029 (14)	−0.0298 (15)

### Geometric parameters (Å, º)

**Table:**

Nd2—O11	2.393 (2)	O11—C5	1.265 (4)
Nd2—O11^i^	2.670 (2)	O16W—H16A	0.8508
Nd2—O5	2.446 (2)	O16W—H16B	0.8501
Nd2—O8^ii^	2.487 (2)	O17W—H17A	0.811 (18)
Nd2—O16W	2.476 (3)	O17W—H17B	0.836 (18)
Nd2—O17W	2.606 (3)	O12—C5	1.268 (4)
Nd2—O12^i^	2.514 (2)	O12W—H12A	0.8534
Nd2—O18	2.502 (2)	O12W—H12B	0.8534
Nd2—O7^iii^	2.456 (2)	O9—C1	1.275 (4)
Nd1—O12W	2.536 (2)	O13W—H13A	0.8513
Nd1—O9^iv^	2.785 (2)	O13W—H13B	0.8508
Nd1—O9	2.383 (2)	O10—C1	1.255 (4)
Nd1—O2^iv^	2.397 (2)	O14W—H14A	0.854 (19)
Nd1—O13W	2.536 (2)	O14W—H14B	0.831 (18)
Nd1—O10^iv^	2.481 (3)	C4—H4A	0.9700
Nd1—O14W	2.592 (3)	C4—H4B	0.9700
Nd1—O1	2.457 (2)	C4—C5	1.502 (4)
Nd1—O4^v^	2.390 (2)	C4—C3	1.523 (5)
S2—O6	1.479 (2)	C1—C2	1.491 (5)
S2—O5	1.485 (2)	C2—H2A	0.9700
S2—O8	1.470 (2)	C2—H2B	0.9700
S2—O7	1.472 (2)	C2—C3	1.516 (5)
S1—O2	1.471 (2)	C3—H3A	0.9700
S1—O1	1.457 (2)	C3—H3B	0.9700
S1—O4	1.449 (3)	O18—H18A	0.827 (18)
S1—O3	1.452 (3)	O18—H18B	0.814 (18)
			
O11—Nd2—O11^i^	68.86 (8)	O8—S2—O6	109.74 (13)
O11—Nd2—O5	85.94 (8)	O8—S2—O5	109.14 (14)
O11—Nd2—O8^ii^	78.84 (7)	O8—S2—O7	111.34 (13)
O11—Nd2—O16W	145.48 (8)	O7—S2—O6	109.63 (14)
O11—Nd2—O17W	140.93 (8)	O7—S2—O5	108.47 (13)
O11—Nd2—O12^i^	118.53 (7)	O1—S1—O2	111.67 (15)
O11—Nd2—O18	73.89 (8)	O4—S1—O2	107.78 (16)
O11—Nd2—O7^iii^	74.65 (7)	O4—S1—O1	109.63 (16)
O5—Nd2—O11^i^	139.33 (7)	O4—S1—O3	109.07 (19)
O5—Nd2—O8^ii^	140.68 (7)	O3—S1—O2	108.79 (16)
O5—Nd2—O16W	99.03 (8)	O3—S1—O1	109.83 (17)
O5—Nd2—O17W	71.78 (8)	Nd2—O11—Nd2^i^	111.14 (8)
O5—Nd2—O12^i^	140.61 (7)	C5—O11—Nd2	156.8 (2)
O5—Nd2—O18	70.81 (7)	C5—O11—Nd2^i^	91.97 (19)
O5—Nd2—O7^iii^	72.67 (7)	S2—O5—Nd2	138.58 (13)
O8^ii^—Nd2—O11^i^	66.58 (7)	S2—O8—Nd2^vi^	130.54 (13)
O8^ii^—Nd2—O17W	137.65 (9)	Nd2—O16W—H16A	122.8
O8^ii^—Nd2—O12^i^	77.46 (7)	Nd2—O16W—H16B	121.4
O8^ii^—Nd2—O18	70.18 (7)	H16A—O16W—H16B	104.6
O16W—Nd2—O11^i^	119.98 (8)	Nd2—O17W—H17A	118 (3)
O16W—Nd2—O8^ii^	75.90 (8)	Nd2—O17W—H17B	111 (3)
O16W—Nd2—O17W	71.46 (9)	H17A—O17W—H17B	107 (3)
O16W—Nd2—O12^i^	78.27 (8)	C5—O12—Nd2^i^	99.3 (2)
O16W—Nd2—O18	75.62 (9)	Nd1—O12W—H12A	109.7
O17W—Nd2—O11^i^	108.23 (8)	Nd1—O12W—H12B	109.0
O12^i^—Nd2—O11^i^	49.67 (7)	H12A—O12W—H12B	104.3
O12^i^—Nd2—O17W	70.17 (8)	Nd1—O9—Nd1^iv^	109.09 (9)
O18—Nd2—O11^i^	126.89 (7)	C1—O9—Nd1^iv^	88.51 (19)
O18—Nd2—O17W	124.49 (8)	C1—O9—Nd1	161.0 (2)
O18—Nd2—O12^i^	142.32 (8)	S1—O2—Nd1^iv^	142.78 (14)
O7^iii^—Nd2—O11^i^	70.19 (7)	Nd1—O13W—H13A	109.5
O7^iii^—Nd2—O8^ii^	135.02 (7)	Nd1—O13W—H13B	109.6
O7^iii^—Nd2—O16W	139.53 (8)	H13A—O13W—H13B	104.6
O7^iii^—Nd2—O17W	68.32 (8)	C1—O10—Nd1^iv^	103.6 (2)
O7^iii^—Nd2—O12^i^	84.15 (8)	Nd1—O14W—H14A	131 (3)
O7^iii^—Nd2—O18	132.77 (8)	Nd1—O14W—H14B	120 (3)
O12W—Nd1—O9^iv^	108.50 (8)	H14A—O14W—H14B	104 (3)
O12W—Nd1—O14W	110.17 (10)	S1—O1—Nd1	144.54 (15)
O9—Nd1—O12W	146.52 (8)	H4A—C4—H4B	107.7
O9—Nd1—O9^iv^	70.91 (9)	C5—C4—H4A	108.8
O9—Nd1—O2^iv^	75.27 (8)	C5—C4—H4B	108.8
O9—Nd1—O13W	141.04 (8)	C5—C4—C3	113.8 (3)
O9—Nd1—O10^iv^	119.29 (8)	C3—C4—H4A	108.8
O9—Nd1—O14W	83.11 (10)	C3—C4—H4B	108.8
O9—Nd1—O1	74.03 (9)	O9—C1—Nd1^iv^	66.63 (18)
O9—Nd1—O4^v^	89.01 (9)	O9—C1—C2	120.3 (3)
O2^iv^—Nd1—O12W	137.40 (8)	O10—C1—Nd1^iv^	52.67 (17)
O2^iv^—Nd1—O9^iv^	70.71 (8)	O10—C1—O9	118.8 (3)
O2^iv^—Nd1—O13W	71.12 (8)	O10—C1—C2	120.8 (3)
O2^iv^—Nd1—O10^iv^	85.99 (9)	C2—C1—Nd1^iv^	167.7 (3)
O2^iv^—Nd1—O14W	73.06 (9)	O11—C5—Nd2^i^	63.04 (17)
O2^iv^—Nd1—O1	136.14 (8)	O11—C5—O12	118.9 (3)
O13W—Nd1—O12W	71.15 (8)	O11—C5—C4	121.6 (3)
O13W—Nd1—O9^iv^	114.29 (7)	O12—C5—Nd2^i^	55.97 (16)
O13W—Nd1—O14W	68.79 (9)	O12—C5—C4	119.5 (3)
O10^iv^—Nd1—O12W	67.24 (8)	C4—C5—Nd2^i^	175.3 (3)
O10^iv^—Nd1—O9^iv^	48.43 (8)	C1—C2—H2A	108.7
O10^iv^—Nd1—O13W	77.67 (9)	C1—C2—H2B	108.7
O10^iv^—Nd1—O14W	144.63 (9)	C1—C2—C3	114.2 (3)
O14W—Nd1—O9^iv^	139.56 (9)	H2A—C2—H2B	107.6
O1—Nd1—O12W	74.58 (8)	C3—C2—H2A	108.7
O1—Nd1—O9^iv^	70.08 (8)	C3—C2—H2B	108.7
O1—Nd1—O13W	144.93 (9)	C4—C3—H3A	108.7
O1—Nd1—O10^iv^	82.52 (10)	C4—C3—H3B	108.7
O1—Nd1—O14W	132.11 (10)	C2—C3—C4	114.2 (3)
O4^v^—Nd1—O12W	70.57 (9)	C2—C3—H3A	108.7
O4^v^—Nd1—O9^iv^	140.98 (9)	C2—C3—H3B	108.7
O4^v^—Nd1—O2^iv^	137.22 (10)	H3A—C3—H3B	107.6
O4^v^—Nd1—O13W	102.45 (9)	Nd2—O18—H18A	110 (3)
O4^v^—Nd1—O10^iv^	135.22 (10)	Nd2—O18—H18B	123 (3)
O4^v^—Nd1—O14W	65.60 (10)	H18A—O18—H18B	107 (3)
O4^v^—Nd1—O1	72.38 (10)	S2—O7—Nd2^v^	141.35 (13)
O6—S2—O5	108.45 (13)	S1—O4—Nd1^iii^	164.56 (18)
			
Nd2—O11—C5—Nd2^i^	−174.9 (5)	O8—S2—O5—Nd2	128.04 (19)
Nd2—O11—C5—O12	−178.5 (3)	O8—S2—O7—Nd2^v^	32.0 (3)
Nd2^i^—O11—C5—O12	−3.5 (3)	O9—C1—C2—C3	−148.9 (3)
Nd2—O11—C5—C4	4.0 (7)	O2—S1—O1—Nd1	−18.1 (4)
Nd2^i^—O11—C5—C4	179.0 (3)	O2—S1—O4—Nd1^iii^	−137.7 (7)
Nd2^i^—O12—C5—O11	3.8 (3)	O10—C1—C2—C3	34.9 (5)
Nd2^i^—O12—C5—C4	−178.6 (2)	O1—S1—O2—Nd1^iv^	31.5 (3)
Nd1—O9—C1—Nd1^iv^	158.3 (7)	O1—S1—O4—Nd1^iii^	−15.9 (8)
Nd1—O9—C1—O10	166.0 (5)	C1—C2—C3—C4	−173.6 (3)
Nd1^iv^—O9—C1—O10	7.7 (3)	C5—C4—C3—C2	−157.3 (3)
Nd1^iv^—O9—C1—C2	−168.6 (3)	C3—C4—C5—O11	−134.7 (3)
Nd1—O9—C1—C2	−10.3 (9)	C3—C4—C5—O12	47.8 (4)
Nd1^iv^—O10—C1—O9	−8.9 (4)	O7—S2—O5—Nd2	6.6 (2)
Nd1^iv^—O10—C1—C2	167.4 (3)	O7—S2—O8—Nd2^vi^	−59.51 (19)
Nd1^iv^—C1—C2—C3	89.6 (11)	O4—S1—O2—Nd1^iv^	151.9 (3)
O6—S2—O5—Nd2	−112.4 (2)	O4—S1—O1—Nd1	−137.5 (3)
O6—S2—O8—Nd2^vi^	62.05 (19)	O3—S1—O2—Nd1^iv^	−89.9 (3)
O6—S2—O7—Nd2^v^	−89.7 (2)	O3—S1—O1—Nd1	102.7 (3)
O5—S2—O8—Nd2^vi^	−179.23 (14)	O3—S1—O4—Nd1^iii^	104.4 (8)
O5—S2—O7—Nd2^v^	152.09 (19)		

Symmetry codes: (i) −*x*+2, −*y*, −*z*; (ii) −*x*+2, *y*−1/2, −*z*−1/2; (iii) *x*, −*y*+1/2, *z*+1/2; (iv) −*x*+1, −*y*+1, −*z*; (v) *x*, −*y*+1/2, *z*−1/2; (vi) −*x*+2, *y*+1/2, −*z*−1/2.

### Hydrogen-bond geometry (Å, º)

**Table:**

*D*—H···*A*	*D*—H	H···*A*	*D*···*A*	*D*—H···*A*
O16*W*—H16*A*···O3^vii^	0.85	1.91	2.709 (4)	155
O16*W*—H16*B*···O6^v^	0.85	1.94	2.774 (3)	165
O17*W*—H17*A*···O12*W*^viii^	0.81 (2)	2.38 (4)	2.966 (4)	130 (4)
O17*W*—H17*B*···O3^vii^	0.84 (2)	2.09 (2)	2.903 (4)	165 (4)
O12*W*—H12*A*···O6^ix^	0.85	2.10	2.825 (4)	143
O12*W*—H12*B*···O17*W*^x^	0.85	2.07	2.904 (4)	166
O13*W*—H13*A*···O12^xi^	0.85	1.95	2.733 (3)	153
O13*W*—H13*B*···O3^xii^	0.85	1.99	2.745 (4)	148
O14*W*—H14*A*···O13*W*^xiii^	0.85 (2)	2.13 (2)	2.966 (4)	165 (4)
O18—H18*A*···O6^ii^	0.83 (2)	2.03 (2)	2.826 (3)	160 (3)
O18—H18*B*···O5^v^	0.81 (2)	2.00 (2)	2.805 (3)	170 (4)

Symmetry codes: (ii) −*x*+2, *y*−1/2, −*z*−1/2; (v) *x*, −*y*+1/2, *z*−1/2; (vii) *x*+1, −*y*+1/2, *z*−1/2; (viii) *x*+1, *y*, *z*; (ix) *x*−1, *y*, *z*; (x) *x*−1, −*y*+1/2, *z*−1/2; (xi) −*x*+1, *y*+1/2, −*z*−1/2; (xii) *x*, *y*, *z*−1; (xiii) −*x*+1, −*y*+1, −*z*−1.
